# Carotid and Vagal Body Paragangliomas

**Published:** 2013-05-06

**Authors:** Luca del Guercio, Donatella Narese, Doriana Ferrara, Lucia Butrico, Andrea Padricelli, Massimo Porcellini

**Affiliations:** 1 Vascular Surgery, Federico II University of Naples, Italy; 2 Vascular Surgery, University of Salerno, Salerno, Italy

**Keywords:** Carotid body tumor, Vagal paraganglioma

## Abstract

Between 1972 and 2012, 25 patients presenting 32 paragangliomas of the neck were observed. Tumor locations included the carotid body (CBTs) in 21 patients and the vagus nerve in 4. Four patients had bilateral CBT and one a bilateral vagal tumor; a metachronous bilateral jugulare paraganglioma was diagnosed in one patient with bilateral CBT Shamblin type III. Five patients presented CBTs type II and three type III. Preoperative embolization was performed in 5 CBTs, with no significant difference in blood loss. Twenty-nine paragangliomas were resected (with three internal carotid artery resection): there were no cerebrovascular accident or perioperative death. Nine patients (36%) had cranial nerve palsy prior to surgery and a postoperative nerve dysfunction occurred in four other tumors (16%). Persistent nerve deficits occurred in 3 patients (12%). No evidence of malignancy was shown, intraoperatively or during a postoperative follow-up period (9 months to 18 years; mean: 8 years).

## I. INTRODUCTION

Paragangliomas of the neck are rare, highly vascular tumors arising from the carotid glomus, jugulare ganglion, vagal nerve body, and other small chemoreceptor organs. Originally described as carotid glomus tumors and later as chemodectomas, they were subsequently allocated to the APUD tumor group, though generally non functioning (non-chromaffin paragangliomas). Carotid body tumors (CBTs) are the most common paraganglioma [ 1 ] , with an incidence of about 1:30,000 in the general population, they account for >50% of head and neck paragangliomas, while vagal paragangliomas account for 5%. The carotid body consists of chemoreceptors that aid in homeostasis by increasing the ventilator rate when stimulated with changing blood concentrations of oxygen, carbon dioxide, and pH. Typically, these tumors manifest between the third and sixth decades of life and affect women five times as often as men. The incidence of these tumors is increasing in population living at high altitudes [ 2 ] and who are affected by chronic obstructive pulmonary disease, so that it was hypothesized that chronic hypoxia might be a cause [ 3 ] . They may have a familial occurrence (up to 50%), and bilateral and multiple tumors are found mostly in familial cases than in sporadic cases. Familial forms are mostly associated with germline mutations [ 4 ] of genes encoding succinate dehydrogenase (SDHB, SDHC, SDHD), and more recently, of SDHAF2 and TMEM127 genes. Epidemiological studies have shown a particularly high incidence of these genetically determined forms of the disease in some endemic areas, including the Trentino region in Italy [ 5 ] . Generally benign, they are slow-growing but may progress locally, damage the lower cranial nerves, and involve carotid vessels, with the onset of symptoms [ 6 ] or develop malignant characteristics (< 10% of all paragangliomas) [ 7 ] . The unsatisfactory results of surgical treatment encountered in the past [ 8 ] are of historical interest, and today surgical excision at an early stage is the treatment of choice to prevent further growth with the risk of involvement of adjacent neurovascular structures. This trend has led to a decrease in mortality and surgery-induced morbidity. The contemporary interest in these tumors regards early diagnosis, identification of the best technique to avoid surgical damage in the cranial nerves (10–30%), the role of preoperative embolization and of radiotherapy.

## II. MATHERIALS AND METHODS

Between 1972 and 2012, 25 patients with 32 cervical paragangliomas were observed. Patients consisted of 18 men and 7 women, and their age ranged from 18 to 71 years (median, 38 yr).

The most common site was the carotid glomus (21 patients), and vagal nerve (4 patients). Four patients had bilateral CBTs and one a bilateral vagal paraganglioma: four of these underwent staged excision. Three patients had previously undergone contralateral cervical paraganglioma at another institution. A metachronous bilateral jugulare paraganglioma was incidentally found in one patient with bilateral Shamblin type III CBT.

The most frequent symptom was a neck mass in 19 patients (76%) and cranial nerve deficit in 9 patients (36%). A number of patients presented with multiple symptoms. ( Table I ).

Conventional angiography was the only preoperative imaging modality used in the early years in our series (9 patients), but is now rarely used and only for preoperative embolization, when needed.

During the second half of the observation period, ultrasound scan was applied in 16 patients, CT in 7 and MRI in 4 patients.

The investigations undertaken in these patients are listed in Table II .

Preoperative embolization was performed 24 h before the tumor resection. There were no significant differences in blood loss between the non-embolization and pre-embolization groups (255±48 mL versus 320± 85 mL; *NS* ). No cerebrovascular accident or death occurred in either groups.

**Fig. 1.**
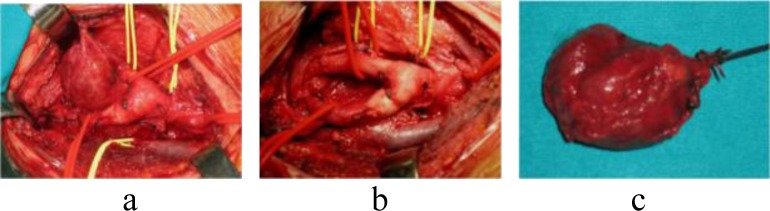
CBT Shamblin type I: caudal-cranial excision (a), and post-resection splayed carotid bifurcation (b). Photograph of excised CBT (c).

Twenty-nine of 32 tumors were excised from the 25 patients; four patients underwent staged bilateral CBT resection. Seventeen CBTs removed, using as general principle a caudocranial approach ( Fig. 1 ), were Shamblin’s type I, five type II, and three type III.

**Fig. 2.**
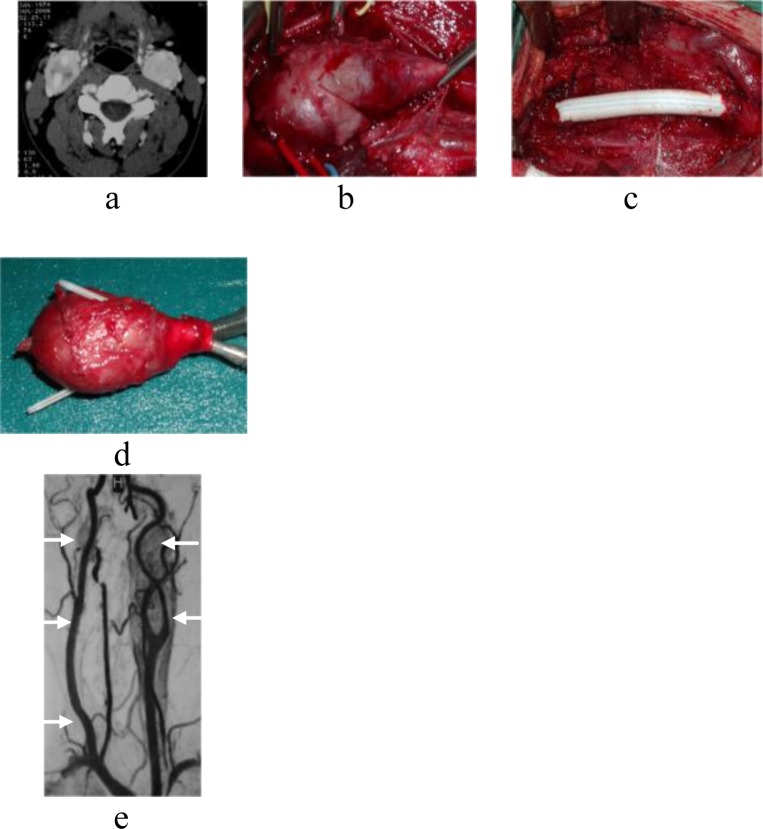
TC shows bilateral CBT Shamblin type III, surrounding carotid vessels (a). Surgical removal of right tumor (b), with ePTFE interposition graft (c). Photograph of the resected tumor (d). RMI (11 months later) showed right carotid reconstruction and a left CBT with concomitant bilateral jugulare tumor (arrows). Left CBT excision and CyberKnife radiosurgery for jugulare tumors (e).

In type III patients, after resection of carotid arteries, the internal carotid was reconstructed by an interposition ePTFE graft, while external carotid artery was ligated. ( Fig. 2 ).

**Fig. 3.**
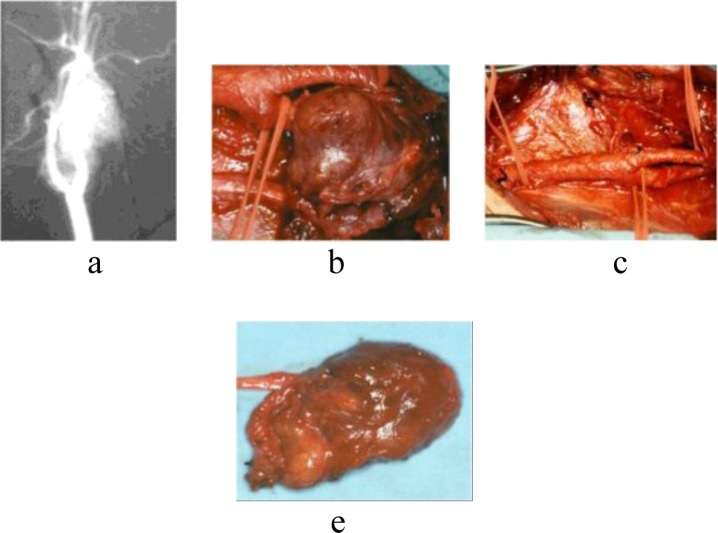
Vagal paraganglioma. MRI showing hypervascular mass dysplaying both ECA and ICA anteriorly (a). Intraoperative photograph of tumor excision (b), with no spreaded carotid bifurcation (c). Photograph of the resected gross specimen shows the mass with no vagal nerve preservation (e).

During the excision of the four vagal paragangliomas, also a segment of the nerve trunk was resected ( Fig. 3 ).

Intraoperative carotid clamping was required in 5 patients. There were no local invasion and locoregional metastases. No blood transfusion was needed.

Tumor removal lead to paresis of the glossopharyngeal nerve (n=1), the vagal nerve (n=1), the hypoglossal (n=1) and sympathetic nerves (n=1). These additional cranial nerve injuries occurred in one Shamblin’s type II patient and in the three Shamblin’s type III patients undergoing carotid artery resection. Follow-up varied from 9 months to 18 years (mean: 8 years). Among the patients with nerve injuries 10 out of 13 (76.9%) healed after an average 7 months; the remaining cranial nerve lesions did not resolve, with a total of 3 patients (12%) with persistent cranial nerve damage. No local recurrence or malignancy occurred during follow-up. CyberKnife stereotactic radiotherapy was used in one our patient with bilateral glomus jugulare tumor after Shamblin type III bilateral CBT resection, with a decrease in tumor size. A conservative approach was used for a contralateral vagal paraganglioma in a high risk 71-years-old patient.

Two patients died of cardiac failure 7 and 13 years after surgery and another of chronic respiratory failure after 14 years.

## IV. DISCUSSION

This is a series of patients with cervical paragangliomas (25 patients, 29 tumors were resected) who have been managed during a 40-year period: CBT represent the most common type of paragangliomas (78.1%) and 21.8% were bilateral. A significant number of cervical paragangliomas in our series are relatively asymptomatic, with a painless lump in the neck, as has been reported by others [ 6 ] . If untreated, these slow-growing tumors tend to compress or surround both the internal (ICA) and the external (ECA) carotid arteries, the adjacent cranial and symphathetic nerves as well as the internal jugular vein (IJV).

During their growth, in the advanced stage the swelling, which has become visible or palpable, is painful and is more easily displaced laterally than vertically (Fontaine’s sign). Other symptoms include headache, dysphonia, disphagia or Horner syndrome occasionally. A systolic murmur may be noted but hypertension is rarely encountered. Although these may be considered APUD tumours, and later a part of DNES (Diffuse Neuroendocrine System), they are rarely functioning. Differential diagnosis includes branchial cyst, primary or metastatic lymphadenopathy, salivary gland or cranial nerve tumours, as well as carotid artery aneurysms. Shamblin et al. proposed the classification of CBTs based on the involvement of carotid vessels in 1971 [ 9 ] . Shamblin group I tumors are localized and not involve the major vessels, group II are adherent or partially surround the vessels, and group III are large and encase the vessels. The size of tumor is positively correlated to the Shamblin classification because CBTs become more adherent to carotid vessels as they become larger.

Ultrasound examination demonstrates a heterogeneous, hypoechoic, hypervascular mass with splaying of carotid bifurcation. [ 10 ] .

Conventional angiography or Digital subtraction angiography remains helpful to demonstrate the vascular supply (feeding vessels and collateral supply) of paraganglioma, the relationship of the mass to the ICA and IJV, its patency or occlusion in larger paragangliomas. Early in ours series, angiography was used routinely before undertaking tumor resection; today conventional angiography, in ours hands, is performed only for preoperative embolization. Carotid body tumour has a characteristic “lyre” appearance, while vagal paragangliona is generally situated more superiorly and displaces both external and internal carotid arteries anteromedially. Modern CT or MRI can provide definitive imaging, including information about the vascular nature of the tumour, the tumour blood supply, involvement of ICA, as well any intracranial infiltration, and the presence of contralateral paraganglioma [ 11 ] . Functional imaging is performed to detect multiple lesions. Octreoscan ( ^111^ indium) is the reference modality. [ 12 ] , but ^18^ FDOPA PET or ^18^ F-FDA PET/TC are also very reliable.

Genetic testing must be performed in all patients looking for a SDHx gene mutation. Blood and urine metanephrine must be evaluated in hypertensive patients, and were normal in three ours hypertensive patients.

Cervical paragangliomas are rarely subjected to open or fine needle aspiration cytology (FNAC) due to high risk for procedure-related bleeding [ 13 ] .

Once diagnosed, cervical paragangliomas should to be removed because their development will involve contiguous structures, with difficult excision and postoperative cranial nerve damage. The difficulty of excision can be assessed only intraoperatively. Attempts to establish preoperative relationship between tumour dimensions and surgical difficulty have been done: carotid paragangliomas over 4–5 cm surround partially or completely carotid vessels and produce higher incidence of complications [ 14 ] .

Surgical excision of the CBT includes proximal and distal control of the carotid arteries with ligation of feeding branches from the ICA and dissection of the mass starting at the carotid bifurcation and continuing in a caudal-cranial fashion. Elective ECA ligation can be helpful for tumor mobilization, but the hemostatic effect of proximal ECA ligation is still controversial [ 15 ] .

Alternatively, the dissection of the tumor can be started cranially [ 16 ] . This modified approach facilitates early proximal control over the majority of adjacent nerves, (VII, IX, X, XII, and especially the superior laryngeal nerve) reducing the risk of postoperative morbidity. In our experience, the caudo-cranial approach is preferred and results in a low risk to the vessels and minimal bleeding [ 17 ] . In case of Shamblin type III carotid body tumor, excision of the entire carotid bifurcation with ligation of the ECA and interposition of a vascular conduit (saphenous vein, ePTFE) for the ICA might be necessary. In ours three type III CBTs, after resection of carotid arteries, the ICA was reconstructed by an interposition ePTFE graft, while ECA was ligated. On small tears, primary closure or patch angioplasty can be performed to repair them.

Temporary carotid clamping may be useful to reduce intraoperative blood loss, and was used in five of ours patients; during ICA clamping, carotid shunt can reduce the risk of stroke [ 18 ] . During the excision of the ours four vagal paragangliomas, a segment of the nerve trunk at the nodose ganglion was resected with no further functional impairment of the vagal nerve.

In our patients, postoperative cranial nerve deficits developed in four patients (16%), and they belonged to one Shamblin type II and three CBTs type III.

There has been controversy concerning the usefulness of preoperative embolization. Some authors prefer routine preoperative embolization because it can lower blood flow and decrease tumour size, particularly in larger tumors (Shamblin type II and III). Others disagree on preoperative routine embolization due to postembolization morbidity such the potential risk of stroke by embolic particles. In addition, preoperative embolization does not decrease rates of cranial nerve injuries, although most are temporary [ 19 ] . In our series, there was no significant difference in intraoperative blood loss and operation time between the embolization (5 tumors) and nonembolization groups. In addition, preoperative embolization by direct percutaneous intratumoral injection of cyanoacrylate glue [ 20 ] or Onyx ^R^ [ 21 ] , as well as devascularization by covered stent placement [ 22 ] . in the ICA have been reported. We believe that the degree of invasion of tumors to the wall of carotid vessels and surgeon’s experience might be more important factors for intraoperative bleeding than the effects of embolization.

Some authors have found that radiotherapy is effective in inhibiting further growth of CBTs [ 23 ] . However, it is often considered to be an alternative treatment modality for patients who cannot undergo surgery due to extensive involvement, multiple tumors, and high operative risk. In dopamine-secreting CBTs radiotherapy has been reported to be efficacious for reduction in tumor size and progressive decline and eventual normalization of urinary dopamine excretion [ 24 ] . Gamma knife radiosurgery and CyberKnife stereotactic radiotherapy [ 25 ] provide a higher degree of accuracy and precision than conventional radiotherapy, as in our patient presenting bilateral glomus jugulare tumor after Shamblin type III bilateral CBT resection.

## V. CONCLUSIONS

In conclusion, Shamblin III CBTs have a high risk of neurological complications. Therefore, early detection by modern imaging modalities and prompt surgical resection will decrease surgical morbidity.

Preoperative embolization of CBTs does not lead to a significant reduction in intraoperative blood loss in our study cohort.

## Figures and Tables

**TABLE I:** PRESENTING SYMPTOMS FOR 32 CERVICAL PARAGANGLIOMAS (IN 25 PATIENTS)

	No (%)
Mass	19 (76)
Pain	8 (32)
Vertigo	3 (12)
Dysphonia/mild dysphagia	5 (20)
Hoarseness	4 (16)
Cranial nerve palsy	9 (36)
Bruit	7 (28)
Hypertension	3 (12)

**TABLE II:** DIAGNOSTIC MODALITIES USED IN THE DIAGNOSIS OF 32 CERVICAL PARAGANGLIOMAS

	No (%)
Angiography	9 (28.1)
Computed tomography angiography	7 (21.8)
Magnetic resonance angiography	4 (12.5)
Ultrasound imaging	16 (12.0)
